# Tannic and gallic acids alter redox-parameters of the medium and modulate biofilm formation

**DOI:** 10.3934/microbiol.2019.4.379

**Published:** 2019-12-27

**Authors:** Zoya Samoilova, Alexey Tyulenev, Nadezhda Muzyka, Galina Smirnova, Oleg Oktyabrsky

**Affiliations:** Laboratory of Physiology and Genetics of Microorganisms, Institute of Ecology and Genetics of Microorganisms, Russian Academy of Sciences, Perm Federal Research Center, Perm, Russia

**Keywords:** tannic and gallic acid, mutations in biofilm formation, *pgaA*, *csgA*, SOS-response, *Escherichia coli*

## Abstract

Tannic (TA) and gallic (GA) acids are known to have both anti- and prooxidant properties however recently they have been described as potential anti-biofilm agents although their mechanisms of action on bacterial cells remain obscure. The aim of our research was to elucidate the role of prooxidant actions of these plant phenolic compounds in bactericidal effects and biofilm formation. In our experiments, both compounds demonstrated strong oxidative properties that altered activity of stress regulons and contributed to decrease of CFU and ability of cells to maintain membrane potential. Stimulation of biofilm formation was observed in all the strains with the exception of the strains deficient in flagella synthesis. Both compounds demonstrated bactericidal effect which was weakened in biofilms. TA efficiently killed bacteria in the bioflms of *pgaA* mutant which pointed out an important role of poly-beta-1,6-N-acetyl-D-glucosamine (PGA) polysaccharide in matrix formation. Similar effects of TA in *recA* mutant indicate involvement of SOS-response into reaction towards exposure with TA. Gallic acid-induced killing was more pronounced in the biofilms of *csgA* mutant revealing role of curli in protection against GA toxicity.

## 1. Introduction

Investigation of bacterial biofilms is a relevant topic both in fundamental and applied sciences. The general concept of biofilm production is quite well understood (bacterial surface attachment, formation of biofilm, dispersal), however regulatory processes of motile-sessile transitions still need to be elucidated. Furthermore, the rise of antibiotic resistant biofilm producing strains motivated a search for compounds that might effectively block formation of biofilms [1]. Plant polyphenolic tannic acid (TA) and its monomer gallic acid (GA) have been recently reported as potentially strong anti-biofilm agents [2]–[5]. Despite numerous researches, mechanisms of bactericidal anti-biofilm action remain obscure because existing works present data on MICs (mimimal inhibitory concentrations) and MBCs (minimal bactericidal concentrations) in planktonic cultures and mass biofilm formation values in the presence of GA and TA. But in fact, under these conditions, there is very little information on both viability of cells inside the biofilms and effects on the genes involved. In Gram-negative bacteria *Escherichia coli* attachment to solid surfaces involves *csgBAC* and *csgDEFG* operons (synthesis, secretion, and assembly of curli components) under certain conditions [6],[7]. Production of biofilm matrix main exopolysaccharides poly-beta-1,6-N-acetyl-D-glucosamine (PGA) and colanic acid is controlled by *pgaABCD* and *cps* operons [8],[9]. Regulation of these processes requires global stress response regulator RpoS which increases levels of signal molecule known as bis-(3′-5′)-cyclic diguanylic acid (c-di-GMP), stimulating loss of flagella, expression of curli, cellulose and PGA synthesis [10]. It has recently been shown that ci-di-GMP signaling in bacterial biofilm formation is redox-sensitive [11],[12]. Plant phenolic compounds are known as redox active substances with antioxidant and prooxidant properties [13] that may act as stimulants for normal gut microbiota growth and development contributing to improvement of state of human health [14],[15]. Fundamental mechanisms of TA and GA on biofilm formation are still poorly understood while dualism of plant phenolics action (pro- and antioxidant activities) requires a diligent research involving model microorganisms. Using laboratory strains originated from *Escherichia coli* BW25113 and its mutant derivatives which genetics and physiology under normal conditions are quite well understood allows observing and interpreting both possible modes of action of TA and GA. In order to check if redox properties of these compounds make any contribution to biofilm formation, we measured their real-time effects on redox parameters including dissolved oxygen (dO_2_), medium redox potential (Eh) and sulfide levels. Earlier, using *E. coli* BW25113 we have shown that 1 mg/ml TA and 4 mg/ml GA acted as MICs on M9 media with glucose, no MBC or MBPC for TA was found, but for GA these were equal to 4 mg/ml [16]. In this paper, we studied the effects of sub-MICs of TA and GA on colony-forming ability of biofilms and their counterpart planktonic cultures, mass and specific biofilm formation in wild-type *E. coli* BW25113 strain and mutants lacking genes involved in surface attachment (*csgA*, *ydeH*), exopolysaccharide production (*pgaA*, *wcaM*), general stress response and SOS-response (*rpoS*, *recA*) as well as estimated effects of TA and GA on changes in membrane potential (ΔΨ) and extracellular potassium (K^+^), expression of *sulA::lacZ* and *katG::lacZ* genes.

## 2. Materials and methods

### 2.1. Strains and growth conditions

The strains of *E. coli* BW25113 (wt) and its derivatives JW1010 (*ΔpgaA*), deficient in production of exopolysaccharide poly-beta-1,6-N-acetyl-D-glucosamine (PGA), JW1025 (*ΔcsgA*), deficient in curli production, JW1528 (*ΔydeH*) with suppressed flagella synthesis, JW2028 (*ΔwcaM*), deficient in production of exopolysaccharide colanic acid, JW2669 (*ΔrecA*), lacking a SOS-response activator, and JW5437 (*ΔrpoS*), deficient in regulator of global stress response, were from Keio collection [17].

The strains carrying transcriptional gene fusions *katG::lacZ* were constructed by transformation of the parental strain with pKT1033 plasmid [18]. The strains with *sulA(sfiA)::lacZ* fusions were created by P1 transduction from the strain DM4000 [19].

Bacteria were grown overnight in M9 minimal medium supplemented with 2 g/L glucose [20]. After centrifugation, the cells were resuspended in fresh M9 medium (4 g/L of glucose) to an initial optical density at 600 nm (OD_600_) of 0.1. This culture was transferred to 96-well polystyrene microtitre plates (200 µL per well) containing 2 mg/mL of GA or 0.5 mg/mL of TA (sub-MICs as preliminary determined) and incubated statically at 37 °C for 22 h to obtain biofilms. These plates were used further to estimate cell viability in biofilms and their counterpart planktonic cultures as well as to measure mass and specific biofilm formation in the presence of TA and GA.

Growth conditions for real-time monitoring of medium redox potential (Eh), dissolved oxygen (dO_2_) and extracellular sulfide and K^+^ levels and for measurement of TA and GA effects on membrane potential and *katG* and *sulA* gene expression were as follows. Night cultures of the wild-type strain were grown with shaking (150 rpm) in M9 minimal glucose (0.15%) medium [20]. After centrifugation, these cells were resuspended in 100 ml of fresh medium (OD_600_ of 0.1) and grown aerobically at 37 °C to OD_600_ of 0.4. Then, TA or GA were added to a final concentration of 1.7 mg/mL and growth was monitored for 1 h. The specific growth rate (µ) was calculated by equation µ = Δln OD_600_/Δt, where t is the time in hours. Phenolic compounds for these experiments were prepared in 96% ethanol and then poured in the ratio 1:100 into 100 ml of the growing cultures in order to exclude effects of the solvent. No significant effects of the solvent were registered then.

Preliminary to the whole set of experiments, pH of the cultivation medium was equal to 7.0. Addition of TA and GA to the cell-free medium to reach the level of working doses both in real-time and microtiter experiments did not cause any pH shifts.

Reagents including gallic acid (catalogue number G7384-100G) and tannic acid (catalogue number 16201), agar, Luria-Bertani broth, 2-nitrophenyl-β-D-galactopyranoside (ONPG), ΔΨ-sensitive fluorescent dye DiBAC4 (3) were from Sigma-Aldrich Chemical Co (St Lous, MO, USA). Other reagents were of analytical grade (Reachim, Russia).

### 2.2. Study of tannic and gallic acids effects on colony-forming ability

Colony-forming ability (CFU/mL) was estimated in planktonic cultures that had developed above the biofilms after 22 h incubation. 10 μL drops of serial dilutions were plated on LB-agar (1.5%). To estimate CFU in biofilms, medium was removed, biofilms were washed with sterile saline and sonicated by two pulses (37 kHz, 30W) for 1 min each with pause time of 1 min in a water bath sonicator (Ultrasonic cleaning unit Elmasonic S10 H, Elma, Germany). Then, OD_600_ was measured and 10 μL drops of serial dilutions were plated on LB-agar. Colonies were counted in 24 h after incubation at 37 °C [21].

In order to estimate effects of TA and GA on viability we used a parameter of total amount of CFU × 10^5^/mL which was the sum of CFU × 10^5^/mL in biofilms and their counterpart planktonic cultures. We also evaluated the percentage amount of CFU × 10^5^/mL in biofilms in relation to the total amount of CFU × 10^5^/mL.

### 2.3. Biofilm formation assay

Mass biofilm formation (BF) was monitored using the modified crystal violet microplate biofilm assay previously described [22],[23]. Wells of 96-well polysterene microtiter plates were prepared as described above. Control wells contained bacteria-free medium or phenolic compounds only. Broth was removed and wells were rinsed twice with 200 µL of sterile saline. The wells were air dried and 150 µL per well of 0.1% crystal violet solution was added for 30 min. Then, the colourant was discarded and the wells were rinsed five times with distilled water. The plates were air dried for 1 h. To quantify biofilms, 200 µL of 96% ethanol was pipetted into each well. After 5 min, 125 µL of the solution was transferred to a separate plate where the OD_540_ were measured using xMark™ Bio-Rad spectrophotometer.

The total biofilm formation (BF) or, the mass of biofilms, was calculated using the formulae:

BF = AB – CW, where AB is the OD_540_ of stained biofilms and CW is the OD_540_ of stained control wells.

To determine specific biofilm formation, values of BF were divided by the CFU values in the biofilms.

### 2.4. Real-time monitoring of dissolved oxygen (dO_2_), medium redox potential (Eh), extracellular potassium (K^+^) and sulfide levels

Dissolved oxygen (dO_2_) in *E. coli* BW25113 cultures were continuously measured directly in the flasks using a Clarke oxygen electrode InPro 6800 (Mettler Toledo). The dO_2_/pH controller of a BioFlo 110 fermentor (New Brunswick Scientific Co., USA) was used for data recording.

Redox potential (Eh) in the cell-free medium and *E. coli* cultures was continuously measured directly in the flasks using platinum and reference electrodes and Mettler Toledo SevenCompact™ pH/Ionmeters S220.

Changes in the levels of extracellular K^+^ were continuously registered directly in the flasks using the system of K^+^-selective (ELIS-121K) and reference electrodes and a computer pH/ion meter cpX-2 (IBI Pushchino, Russia). For K^+^ measurements, *E. coli* cells were grown as described above, except that the medium contained a low K^+^ concentration (0.1 mM).

Extracellular sulfide levels were detected directly in the flasks using the system of sulfide-specific ion-selective XC-S2-001 (Sensor Systems Company, Russia) and reference electrodes and a computer pH/ion meter cpX-2 (IBI Pushchino, Russia).

### 2.5. Evaluation of gallic acid and tannic acid effects on membrane potential

Changes in the membrane potential (ΔΨ) were evaluated using ΔΨ-sensitive fluorescent dye DiBAC4 (3) [24] as described previously [20]. After addition of 1.7 mg/mL phenolic compounds into 100 mL of aerobically growing cultures at OD600 = 0.4 aliquots of culture samples were collected at time 0, 30 and 60 min. Samples of log-phase cells treated with protonophore carbonylcyanide m-chlorophenylhydrazone (CCCP, 20 mM) were used as positive control. Fluorescent cells were counted using a Leica DM2000 microscope as earlier described [25]. Total cell number was counted in transmitted light. About 1000 cells were counted for every sample and all experiments were conducted 3–6 times on separate days.

### 2.6. Determination of β-galactosidase activity

β-galactosidase activity in reporter strains carrying fusions of the genes *katG*, and *sulA* with the gene *lacZ* using a SmartSpec Plus Spectrophotometer (Bio-Rad, USA) was measured [20].

After addition of 1.7 mg/mL polyphenols into 100 mL of aerobically growing cultures at OD_600_ = 0.4 aliquots of culture samples were collected at time zero and further every 15 min during 1 h. β-galactosidase activity was expressed in Miller units, calculated using the formula:

$$\left({\text{OD}}_{\text{42}0}-\text{1}.\text{75}\times {\text{OD}}_{\text{55}0}/\Delta {\text{OD}}_{\text{6}00}\times \text{t}\right)\times \text{6}000$$

where OD_420_ and OD_550_—optical density of the samples, ΔOD_600_ is the difference between OD_600_ of bacterial culture and OD_600_ of bacteria-free medium, t—duration of exposition with ONPG, 6000—coefficient taking into account dilution of the culture.

### 2.7. Statistical analysis of the data

Each result is indicated as the mean value of at least five independent experiments ± the standard error of the mean (SEM). Significant difference was analyzed by Student's *t*-test. A *P*-value of 0.05 was used as the cut-off for statistical significance. Results were analyzed by means of Statistica 6 (ver. 6, 2001; StatSoft Inc.).

## 3. Results

### 3.1. Effects of gallic and tannic acids on CFU of planktonic cultures and biofilms

**Figure 1.**
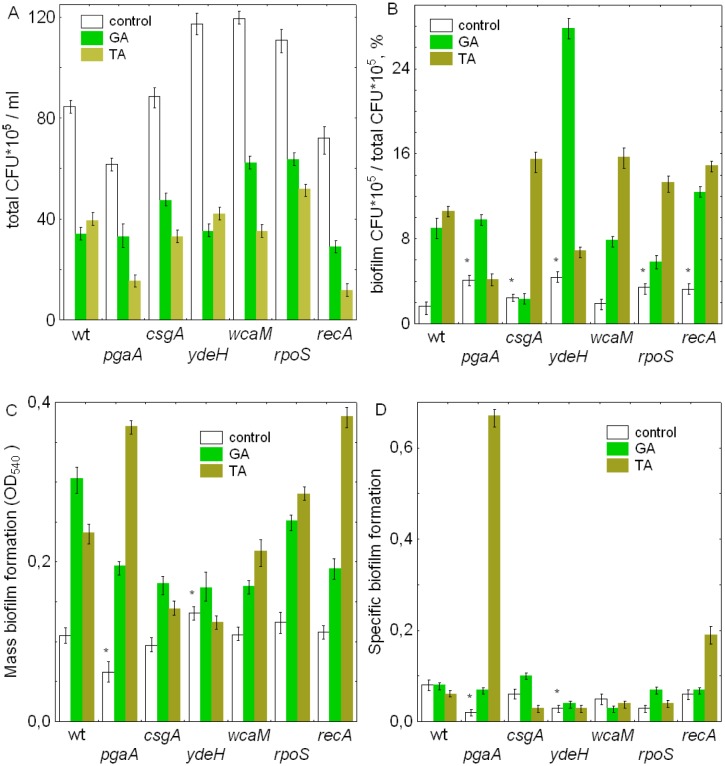
Total colony-forming units (CFU) in *E. coli* strains (A); Share of biofilms in total CFU (%) (B); Mass biofilm formation in *E. coli* strains (C); Specific biofilm formation, OD_540_/CFU in biofilms, in *E. coli* strains (D), after 22 h incubation without phenolic compounds (control), with 2 mg/ml gallic acid (GA), with 0.5 mg/ml tannic acid (TA). *—indicates statistically significant difference from the wild-type strain, P > 0.05.

Without phenolic compounds, after 22 h of static incubation of the wild-type cultures total amount of CFU × 10^5^/mL was equal to 85 ± 10 (Figure 1A) and contained 1.64% of biofilm (Figure 1B). In the presence of TA and GA, total viability decreased by 2 times. However, percentage amount of CFU in biofilms increased up to 11 and 9%, respectively. This showed both strong bactericidal effects on planktonic cultures and an increase in share of CFU in biofilms which could be seen as an adaptive response of the wild-type cells to action of the tested polyphenols.

In the absence of tested compounds, total amount of CFU × 10^5^/mL in *csgA* and *recA* mutant strains was similar to the parental strain. In *ydeH*, *wcaM* and *rpoS* it was about 30% higher while in *pgaA* it was 19% lower compared to the parental strain (Figure 1A). Similarly to the parental strain, incubation with TA and GA led to a decrease in total CFU in all mutant strains. More remarkable effects were found in *recA* and *pgaA* where TA decreased total CFU by 6 and 5 times, respectively, while in the parental strain TA inhibited total CFU only by 2 times (Figure 1A). As for GA, inhibition level of total CFU was quite similar in parental and mutant strains.

Without phenolic compounds, percentage of CFU in biofilms in relation to total CFU increased in all tested mutants with the exception of *wcaM* mutant (Figure 1B). The highest share of biofilms was found in *ydeH* which was 2.6 times higher compared to the wild-type. 22 h incubation with polyphenols increased this parameter in all mutant strains with the exception of GA in *csgA* and TA in *pgaA* strains (Figure 1B).

### 3.2. Effects of gallic and tannic acids on mass and specific biofilm formation

Under our conditions, after 22 h incubation of wild-type bacterial cultures in M9 medium with addition of glucose in 96-microtiter polystyrene plates value of OD_540_ was equal to 0.108 ± 0.009. *pgaA* mutation decreased mass biofilm formation (BF) almost by half. While *ydeH* mutation resulted in 25% BF increase (Figure 1C). Presence of GA and TA resulted in BF stimulation in the wild-type by 2.8 and 2.2 times, respectively. Stimulating effects by both phenolic compounds were observed in all the mutants tested with the exception of *ydeH*. The highest degree of stimulation was seen in *pgaA* where there was a 6-time and 3-time stimulation by TA and GA, respectively, compared to *pgaA* mutant not treated with phenolics.

In the absence of tested compounds, SBF in the wild-type was equal to 0.080 ± 0.008. *pgaA* mutation led to a remarkable decrease of the parameter by 4 times. *ydeH* and *rpoS* mutations decreased SBF by about 2.7 times (Figure 1D). Incubation of the wild-type with both polyphenols had a little effect on SBF. However, in the presence of TA *pgaA* and *recA* mutants dramatically increased SBF by 10 and 3 times, respectively, compared to the wild type treated with TA. *csgA* mutant in the presence of GA demonstrated a slight increase in SBF.

### 3.3. Real-time effects of gallic and tannic acids on the levels of dissolved oxygen (dO_2_), medium Eh, extracellular potassium (K^+^) and sulfide levels

The observed bactericidal action of TA and GA on planktonic cultures after 22 h incubation could result from plant phenolics-induced oxidative stress which stimulated the cells to switch from planktonic lifestyle to biofilms [13]. These metabolic alternations could happen on early stages of incubation of bacterial cultures. So, in a separate series of experiments we decided to observe real time effects of GA and TA on redox-parameters of the cultivation medium.

**Figure 2.**
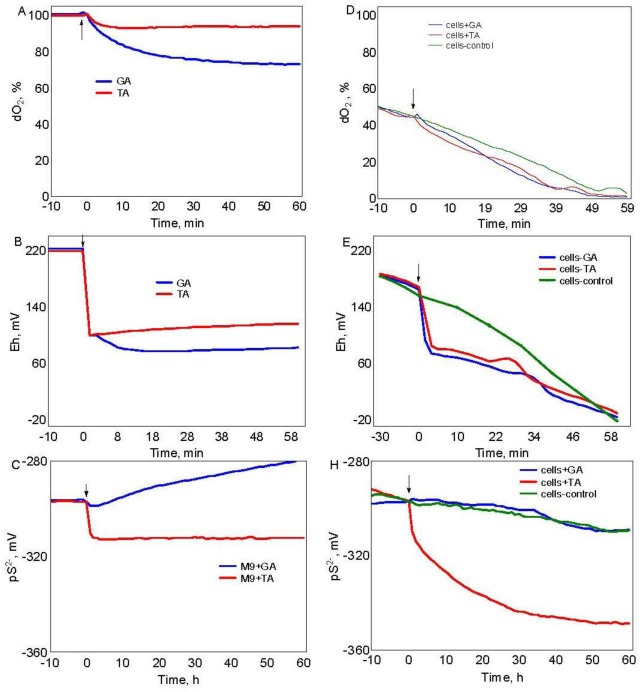
Real-time monitoring of dO_2_ (A), Eh (B) and sulfide levels (C) in cell-free media after addition of 1.7 mg/mL tannic (TA) or gallic (GA) acids; Effects of addition of 1.7 mg/mL tannic (TA) or gallic (GA) acids to aerobically growing wild-type *E. coli* cultures on dO_2_ (D), Eh (E) and sulfide levels (H). Moment of polyphenol addition at time zero is indicated by the arrow.

A set of experiments in cell-free M9 medium allowed us to reveal remarkable levels of redox-activity of GA and TA. Addition of GA and TA into the cell-free M9 medium resulted in gradual decrease of dO_2_ from 100% to 94% and 74% for TA and GA, respectively, revealing autooxidation of these polyphenols (Figure 2A). There also was a sharp and irreversible decrease of cell-free medium Eh approximately by 120 mV (Figure 2B). In cell-free model experiments, we observed a decrease of potential of sulfide-specific electrode by 2 and 18 mV in the presence of GA and TA, respectively. This might reveal presence of small amounts of S^2−^ ions in the tested polyphenols corresponding to concentrations 8 and 35 nM, respectively (Figure 2C).

In real-time experiments the specific growth rate in the wild-type was 0.65 ± 0.02 before addition of tested compounds, while 15 min after addition, TA and GA led to an approximately 3-time decrease in the specific growth rate (data not shown). In microtiter experiments growth rate was not measured.

When TA and GA were added into aerobically growing cultures of *E. coli* BW25113 in mid-logarithmic phase (OD_600_ = 0.4) a slight inhibition of respiration during 15 min was found which then turned to a normal state (Figure 2D). This might point out a response of the cells to a stress provoked by TA and GA.

Without phenolic compounds, cultivation of aerobically growing cultures of *E. coli* BW25113 was accompanied by a gradual decrease of medium Eh up to negative values when oxygen was depleted. When tested compounds were added into aerobically growing cultures in mid-logarithmic phase (OD_600_ = 0.4), there also was a sharp irreversible decrease of medium redox-potential by 97 and 88 mV in the presence of GA and TA, respectively, which further continued till oxygen depletion (Figure 2E).

When TA was added into aerobically growing cultures in mid-logarithmic phase (OD_600_ = 0.4), an irreversible decrease of potential of sulfide-specific electrode by about 52 mV (180 nM of S_2_-ions) was registered during 1h of incubation (Figure 2H) indicating production of sulfide by the cells in response to TA addition.

No significant shifts of extracellular potassium levels were found in the presence of GA and TA (data not shown).

Thus, a sharp drop in Eh after addition of TA and GA and accumulation of sulfide after addition of TA indicate a significant change in redox situation, which might lead to activation of global stress response regulators [10].

### 3.4. Effects of gallic and tannic acids on gene expression

**Figure 3.**
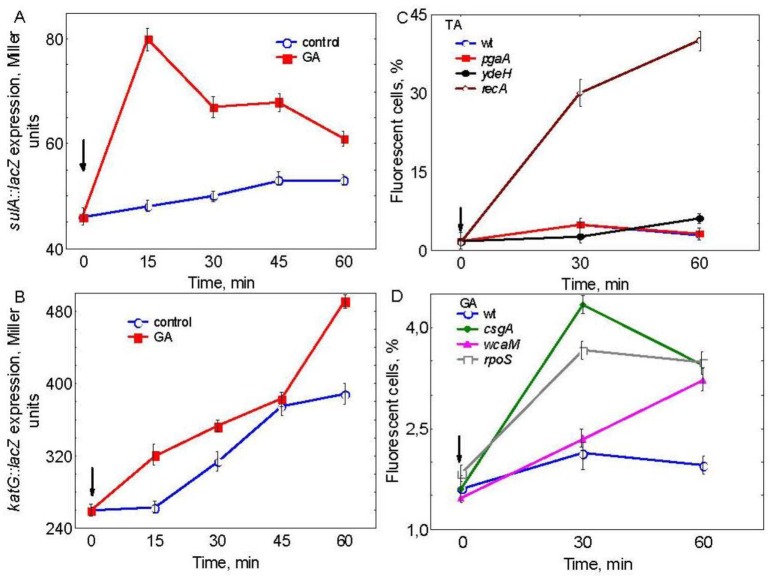
Expression of *sulA::lacZ* (A), *katG::lacZ* (B) in aerobically growing wild-type cultures after addition of 1.7 mg/mL gallic acid (GA); Changes in membrane potential in aerobically growing wild-type cultures after addition of 1.7 mg/mL tannic (TA), (C), or gallic acid (GA), (D). Moment of plant phenolics addition at time zero is indicated by the arrow.

After 15 min of GA addition *sulA::lacZ* expression level sharply increased by about 60% and further remained about 45% higher compared to the cultures not treated with phenolics (Figure 3A). Elevation of expression of gene *sulA* gene can be used as an indicator of DNA damages and a consequent activation of SOS-response [26]. *katG* expression also increased in the presence of GA by about 25% (Figure 3B). Rise in *katG* expression can be used a sign of peroxide stress and activation of oxidative stress response through OxyR regulon [27]. Collectively, these findings revealed contribution of both SOS- and OxyR-regulons in the effects provoked by GA.

### 3.5. Effects of gallic and tannic acids on changes in the membrane potential

Without tested compounds, during 1 h of incubation the wild type and mutant strains showed a similar amount of fluorescent cells with DIBAC (data not shown). TA increased fluorescence in the wild-type by 3 times as well as in *pgaA* and *ydeH* mutants. In *recA* mutant, the increase was 10 times higher compared to the wild-type (Figure 3C). GA did not affect fluorescence in the wild-type during 1 h of incubation. However, it stimulated fluorescence in *rpoS* and *csgA* mutants by about 3 times after 30 min exposure to GA. In case of *wcaM*, 1h after GA addition fluorescence grew up by 2 times compared to the wild-type (Figure 3D).

## 4. Discussion

Previously, we have determined minimum inhibitory concentrations (MICs) for TA and GA which were 1 and 4 mg/mL, respectively, in M9 media with glucose. Interestingly, we had no minimal bactericidal concentration (MBC) and minimal biofilm prevention concentration (MBPC) for TA, while for gallic acid these were 4 mg/mL [16]. These observations revealed distinct effects of the two phenolic compounds on biofilm formation, however redox activity of both is well established showing dual anti- and prooxidant effects [13],[28]. The aim of this work was to elucidate contribution of redox properties of tannic and gallic acids on biofilm formation.

Here, we used sub-MICs of TA and GA to study their effects on biofilms and their counterpart planktonic cultures of the wild-type strain and mutants lacking genes involved in biofilm formation. Incubation of wild-type cultures for 22 h with TA or GA had a bactericidal effect for the plankton but at the same time increased amount of CFU in the biofilms. Taking into account, previously found ability of TA to produce hydrogen peroxide [28] during autooxidation which was also seen here in our real-time monitoring of dO_2_ and Eh, we concluded that an oxidative stress took place and it could contribute to bactericidal action of TA. However, we did not observe elevation of expression of *katG* gene responsible for peroxide stress response during 1h incubation with TA. At the same time, about 3-time decrease of the specific growth rate could stimulate general stress response pathways including biofilm formation [10].

In case of GA we observed a real time increase of *katG::lacZ*, which specifically indicates activation of OxyR regulon in response to peroxide stress [27]. A slight inhibition of respiration and *sulA::lacZ* activation by GA in real-time also showed activation of SOS-response and DNA damage in the presence of GA [26]. Damage of DNA *in vitro* by GA was previously described [29]. These effects of GA could contribute to its bactericidal action. Stimulation of mass biofilm formation and increase CFU in the wild-type biofilms that were found here could be an adaptive strategy to escape from remarkable oxidative activity of both GA and TA [30].

Without phenolic compounds, mass and specific biofilm formation were sensitive to *pgaA* mutation (lack of porin) which in fact blocked release of the main extracellular matrix component - poly-beta-1,6-N-acetyl-D-glucosamine (PGA). However, the amount of CFU in the biofilms increased indicating that cells incapable of PGA synthesis could produce small amounts of biofilm matrix out of other polysaccharides like colanic acid or cellulose [31] and eventually produce a biofilm.

Another important thing to mention is that production of curli might be temperature dependent and is often associated with ambient temperatures. Expression of *csgA* at 37 °C was observed in *E. coli* O157:H7 [7]. In our preliminary experiments we used Congo red binding assay [32],[33] in order to check production of curli at 37 °C in *E. coli* BW25113 and some of its mutant derivatives from the Keio collection. Our results revealed expression of curli in the wild-type. This might sound contradictory as Congo red binding assay was shown to be specific not only to curli production but to other bacterial extracellular features, including cellulose [34]. Therefore, we checked curli expression in the mutant *E. coli* JW1023 (Δ*csgD*), lacking activator of *csgBAC* operon, and *E. coli* JW1025 (Δ*csgA*), lacking a main protein component of curli, and found no binding activity. Collectively, we could conclude that there was production of curli in the wild-type *E. coli* BW25113 at 37 °C.

Effects of plant phenolic compounds on genes associated with biofilm formation are quite poorly described. Therefore, our results might provide some new aspects in this field.

In our conditions, TA could overcome inhibitory effect of *pgaA* mutation on both BF and SBF while GA demonstrated inhibiting effects on BF in *pgaA* mutants which was similar to earlier described suppression of *pgaABC* genes by 0.25 mg/mL GA [2].

In case of *recA* mutant, which had no SOS-response activator, without polyphenols there was no effect on BF, SBF and CFU values. But in the presence of TA, bactericidal action was found which resulted in an increase of SBF. Bactericidal action was also confirmed in the tests with DIBAC where *recA* mutant remarkably increased percentage of fluorescent cells in the presence of TA. TA was reported to inhibit intracellular SOS-response in *E. coli* strains [35] which in our case could contribute to an increased bactericidal action of TA in *recA* mutant. TA and its monomer GA may alter the process of DNA damage *in vitro*, with a higher DNA degrading capacity for GA [36].

Effects of GA on flagella synthesis were studied in *Pseudomonas fluorescens* KM120, where 240 μmol/L GA prevented expression of flagella synthesis genes and reduced colonization [37]. In our case, *ydeH* mutation in *E. coli* implied low levels of c-di-GMP resulting in disruption of flagella synthesis. However, without tested compounds, *ydeH* mutant had both large values of BF and CFU, and, consequently, a decreased value of SBF. In the presence of TA *ydeH* mutant increased BF but decreased CFU (which also coincided with DIBAC test results), increasing SBF. Therefore, TA could also act as bactericidal agent which stimulated biofilm formation as an adaptive response even when flagella were absent.

RpoS is an activator of general stress response in *E. coli* which is involved in stimulation of biofilm formation as a result of slow growth [38]. Mutations in *rpoS* are reported to induce biofilm production even in exponentially growing cultures [39]. In our conditions *rpoS* mutant had BF similar to the wild type but an increased amount of CFU, thus a decreased value of SBF. TA had no effect on BF but increased CFU, diminishing value of SBF. This was consistent with the previously described TA acting on biofilm formation via RpoS-independent pathways [40].

Similarly to *rpoS* mutant, it occurred in *wcaM* mutant, lacking an enzyme for colanic acid synthesis. So, we could suppose TA did not affect pathways of synthesis of colanic acid. On the whole, colanic acid was found to be essential for depth and three-dimensional structure of *E. coli* biofilms but not for surface attachment [41]. Thus, apparently, no significant effects of phenolic compounds on production of biofilms in *wcaM* mutant were observed.

Production of curli is under RpoS control [42]. In our conditions, *csgA* mutant lacking main protein component of curli acted similarly to the wild-type in the absence of phenolic compounds. TA did not stimulate BF or CFU in the biofilms in this strain which could be probably due to the lack of effects of TA on RpoS expression [40]. At the same time, GA reduced both BF and CFU in biofilms, increasing SBF in *csgA*.

## 5. Conclusions

Collectively, our data indicate that TA and GA exhibited strong oxidative properties that altered activity of stress regulons and contributed to decrease of CFU and ability of cells to maintain membrane potential. Both TA and GA stimulated BF in all the strains with the exception of the strains deficient in flagella synthesis. Both phenolic compounds demonstrated bactericidal effect which was weakened in biofilms.

TA efficiently killed bacteria in the bioflms of *pgaA* mutant which pointed out an important role of PGA polysaccharide in matrix formation. Similar effects of TA in *recA* mutant indicate involvement of SOS-response into reaction towards exposure with TA. These observations rise alert when considering TA a potential anti-biofilm agent because situation seems more complicated: a revealed bactericidal effect of TA eventually switched on a strategy to overcome toxic exposure to TA and increase share of viable cells inside the biofilms.

GA-induced killing was more pronounced in the biofilms of *csgA* mutant revealing role of curli in protection against GA toxicity.

In conclusion, our findings indicate that motile-sessile transitions in *E. coli* in the presence of TA and GA are based on dual behaviour of these polyphenols. Their strong oxidative properties switch on pathways that allow persistence to oxidative stress and survival of cells inside biofilms.
